# Selective Targeting and Tissue Penetration to the Retina by a Systemically Administered Vascular Homing Peptide in Oxygen Induced Retinopathy (OIR)

**DOI:** 10.3390/pharmaceutics13111932

**Published:** 2021-11-15

**Authors:** Maria Vähätupa, Niklas Salonen, Hannele Uusitalo-Järvinen, Tero A. H. Järvinen

**Affiliations:** 1 Faculty of Medicine and Health Technology, Tampere University, 33520 Tampere, Finland; maria.vahatupa@tuni.fi (M.V.); niklas.salonen@tuni.fi (N.S.); hannele.uusitalo-jarvinen@tuni.fi (H.U.-J.); 2 Eye Centre & Department of Orthopedics & Traumatology, Tampere University Hospital, 33520 Tampere, Finland

**Keywords:** vascular homing peptide, cell penetrating peptide, angiogenesis, oxygen-induced retinopathy (OIR), retina, diabetic retinopathy, retinopathy of prematurity (ROP), blood-retina barrier, targeted delivery, bystander effect, regenerative medicine, hypoxia, heparan sulphate proteoglycan, bronchopulmonary dysplasia, R-Ras

## Abstract

Pathological angiogenesis is the hallmark of ischemic retinal diseases among them retinopathy of prematurity (ROP) and proliferative diabetic retinopathy (PDR). Oxygen-induced retinopathy (OIR) is a pure hypoxia-driven angiogenesis model and a widely used model for ischemic retinopathies. We explored whether the vascular homing peptide CAR (CARSKNKDC) which recognizes angiogenic blood vessels can be used to target the retina in OIR. We were able to demonstrate that the systemically administered CAR vascular homing peptide homed selectively to the preretinal neovessels in OIR. As a cell and tissue-penetrating peptide, CAR also penetrated into the retina. Hyperoxia used to induce OIR in the retina also causes bronchopulmonary dysplasia in the lungs. We showed that the CAR peptide is not targeted to the lungs in normal mice but is targeted to the lungs after hyperoxia-/hypoxia-treatment of the animals. The site-specific delivery of the CAR peptide to the pathologic retinal vasculature and the penetration of the retinal tissue may offer new opportunities for treating retinopathies more selectively and with less side effects.

## 1. Introduction

Hypoxia driven retinal neovascularization is a key feature in devastating ischemic retinal diseases such as proliferative diabetic retinopathy (PDR) and retinopathy of prematurity (ROP) [1,2]. Abnormal angiogenesis is associated with vascular leakage, hemorrhages and fibrovascular proliferation, which result in retinal edema, retinal/vitreous hemorrhages or tractional retinal detachments and subsequent loss of vision. It is known that the vascular endothelial growth factor-A (VEGF) plays an integral role in causing pathological angiogenesis and vascular leakage [3]. When the cells of the retina experience hypoxia, the transcription factor hypoxia inducible factor-1α (HIF-1α) is stabilized [4,5,6,7,8]. Stabilized HIF-1α translocates to the cell nucleus and forms a complex with HIF-1β. The HIF-1 transcription factor then binds to the hypoxia response element in the genes that promote survival in low-oxygen conditions. The genes that the active HIF-1 signaling triggers in hypoxic cells include large number of angiogenic growth factors, of which VEGF is the most important one [4,5,7]. These growth factors direct the sprouting of the new blood vessel, i.e., angiogenesis, in attempt to deliver oxygen to and address the hypoxia in the tissue [8]. VEGF also induces vascular permeability and leakage, which are evident in ocular NV diseases [4,8]. VEGF-inhibitors have been used with considerable success to inhibit retinal angiogenesis [9,10,11]. However, despite their substantial efficacy they also have their drawbacks [3]. In PDR, the long-term benefits of anti-VEGF have been questioned due to the dramatic loss of the visual field observed after a two-year follow-up [12]. There is also evidence suggesting that the anti-VEGF treatment does not reverse the pathologic process of non-perfusion in DR [13]. Furthermore, anti-VEGFs may lose their therapeutic effect in slowing down the progression of the retinopathy [1,3]. There are also side effects related to the dosing of anti-VEGF compounds. Current anti-VEGF therapeutics are administered by frequent intravitreal injections, which exposes patients repeatedly to a vision threatening complication, endophthalmitis. Frequent injections present a burden for patients and health care service providers, which may result in under treatment and compromise treatment results [10,12,14]. Furthermore, eradication of the neovessels by VEGF inhibitors may worsen the underlying ischemia and drive the formation of new, leaky blood vessels by an alternative molecular mechanism responding to hypoxia [4,8]. The persistency of angiogenic blood vessels leads to the maintaining the hypoxia instead of resolving it [8]. Thus, there is a clear need for alternative therapies.

Furthermore, the treatment of retinal diseases is hindered by the poor bioavailability of the drugs [14,15]. The retina itself poses as a major physical barrier for efficient drug delivery due to poor permeability [14,15]. This is especially relevant for large molecules such as antibodies. The effectiveness of both topical and intravitreal drug applications, in turn, are influenced by drug clearance related to lacrimal fluid and drainage mechanisms, conjunctival systemic absorption, the cornea, and different tissues in the anterior segment of the eye [14,15]. On the other hand, systemic drug administration (as well as the intravitreal administration) is hindered by the blood–aqueous barrier (BAB) and the blood–retina barrier (BRB), resulting in an ocular bioavailability of less than 5% in some cases [14,15,16]. Novel, innovative drug delivery approaches are needed to properly utilize the potential therapeutic value of drugs in retinal disease [14,15,16,17,18].

One of the goals of modern pharmacology is that a drug should be as selective as possible in its activity. It should be active against the disease, while having as few side-effects as possible. This goal is usually obtained by developing molecules that act on a target selectively over-expressed in the disease. However, any drug can be converted into a target organ-specific by a targeted delivery to the desired location. The vascular system provides a platform for this. The expanding understanding of blood vessels on molecular level has shown that each tissue leaves its mark, “a molecular fingerprint” on the lumen of its blood vessels [19,20,21]. These tissue-specific “molecular fingerprints” form a system that is very similar to postal codes. These organ-specific blood vessels can be targeted by systemically administered affinity ligands that seek out, i.e., “home”, to their target by binding to the corresponding vascular zip code, i.e., the receptor on the lumen of the blood vessel [19]. Moreover, various diseases induce the expression of disease-specific molecular signatures on the vasculature, essentially providing a target for the disease specific delivery of systemically administered drugs. This is particularly evident for diseases like cancer and tissue injuries; both of which are associated with tissue hypoxia, which in turn induces new blood vessel growth by angiogenesis [19]. The angiogenic blood vessels are structurally and molecularly different from the dormant blood vessels elsewhere in the body and provide an accessible and abundant target for the organ-specific delivery of therapeutics [19,22].

Oxygen induced retinopathy (OIR) is a widely used experimental disease model for ischemic retinopathies which recapitulates a key pathological feature, the retinal neovascularization [1,23,24]. We decided to explore whether the vascular homing peptide CAR, which recognizes angiogenic vasculature [25,26], can be targeted to pathological retinal neovascularization in OIR after systemic administration. In this study we show that the systemically administered CAR peptide is targeted site-selectively to pathological preretinal neovessels in OIR. Furthermore, we demonstrate tissue penetration to the retina after intravitreally administered CAR-peptide in OIR. As the induction of OIR by hyperoxia also induces bronchopulmonary dysplasia [27], we demonstrate that the systemically administered CAR peptide is not targeted to normal lungs but is detected in the lungs after the OIR induction.

## 2. Materials and Methods

### 2.1. Peptide Synthesis

Peptides were synthesized with an automated peptide synthesizer (Liberty, CEM Corporation, Matthews, NC, USA) by using standard solid-phase fluorenylmethoxycarbonyl chemistry [28]. The peptides were purified by HPLC using 0.1% TFA in acetonitrile-water mixtures to 90–95% purity and validated by Q-TOF mass spectral analysis. During synthesis, the peptides were labeled with 5(6)-carboxyfluorescein (FAM) at the N-terminus using an amino-hexanoic acid spacer as described previously [29]. Both synthesized peptides are cyclic peptides, which form disulfide bonds between the cysteine residues at the end of the peptides.

### 2.2. Mice and Mouse OIR Model

WT and R-Ras KO C57BL/6 mice were used for the study. R-Ras KO mice have been described previously [30]. Before any experiments, R-Ras heterozygous mice were backcrossed eight times with the C57BL/6 strain to obtain homozygous WT and KO mice in the same genetic background. The mice were bred, and the genotype was determined by PCR as described previously [31]. Mice were housed under standard conditions with a 12/12 h dark and light cycle and fed with standard laboratory pellets and water ad libitum. The OIR model was generated as described in detail previously [32,33]. Briefly, the pups at P7 (postnatal day 7) and their nursing mothers were exposed to 75% oxygen (ProOx P110 oxygen controller; Biospherix Ltd., Parish, NY, USA) for 5 days until P12 when they were returned to normal room air.

All animal experiments were conducted under the ARVO Statement for the Use of Animals in Ophthalmic and Vision Research guidelines in accordance with protocols approved by the National Animal Ethics Committee of Finland (protocols # ESAVI/92/04.10.07/2014 and ESAVI/6421/04.10.07/2017).

### 2.3. Peptide Targeting Study

FAM-labelled CAR and *m*CAR peptides were used for the peptide targeting studies as described previously [25,29,34,35]. Peptides were dissolved in PBS. Either healthy mice or R-Ras KO mice at P17 OIR were injected intraperitoneally with peptide solution (5 mg/kg). Two hours after injection, the mice were perfused with Dulbecco’s Modified Eagle Medium (DMEM) containing 1% bovine serum albumin (BSA) while under deep anesthesia

### 2.4. Immunohistochemistry

For immunohistochemistry the eyeballs were harvested, fixed with 4% PFA for 4 h and processed for paraffin embedding. 5 µm thick sections were subjected to antigen retrieval (Tris-EDTA, pH9) and blocked for unspecific binding. To determine the localization of the peptides, paraffin-embedded tissue sections were immunostained with a rabbit anti-fluorescein antibody (#71-1900, Invitrogen, Carlsbad, CA, USA) followed by horseradish peroxidase–labeled anti-rabbit IgG secondary antibody as described previously [25,26,29,34,35]. The peptide localization was then visualized by diaminobenzidine. Sections were counterstained with hematoxylin to visualize the region of cells.

For immunofluorescence (IF) double-stainings of the FAM-labeled peptide and blood vessels, sections were incubated with Isolectin GS-IB_4_ conjugated with Alexa Fluor 594 (#I21413, Invitrogen, Carlsbad, CA, USA). Samples were imaged with a confocal microscope (LSM700, Carl Zeiss, Oberkochen, Germany).

### 2.5. Quantitative Analysis of Immunostaining

All slides were scanned using the NanoZoomer S60 (Hamamatsu Photonics, Hamamatsu City, Japan). Slides were viewed and analyzed using QuPath software version 0.2.0 or later (https://qupath.github.io (accessed on 1 October 2021), Centre for Cancer Research and Cell Biology, Queen’s University Belfast, Belfast, Northern Ireland, UK). Analyzed areas were marked from high magnification images using QuPath. Analysis algorithms were used, and the area of positive staining was determined as described previously [36].

### 2.6. Statistical Analysis

Differences between the groups were statistically tested using the Student’s unpaired and paired *t*-test. The possible difference in the homing was assessed using the log-transformed variables. *p* values of less than 0.05 were considered statistically significant for all tests. The significance level shown refers to two-tailed test.

## 3. Results

### 3.1. CAR Peptide Homes to Preretinal Tufts in OIR

**Figure 1:**
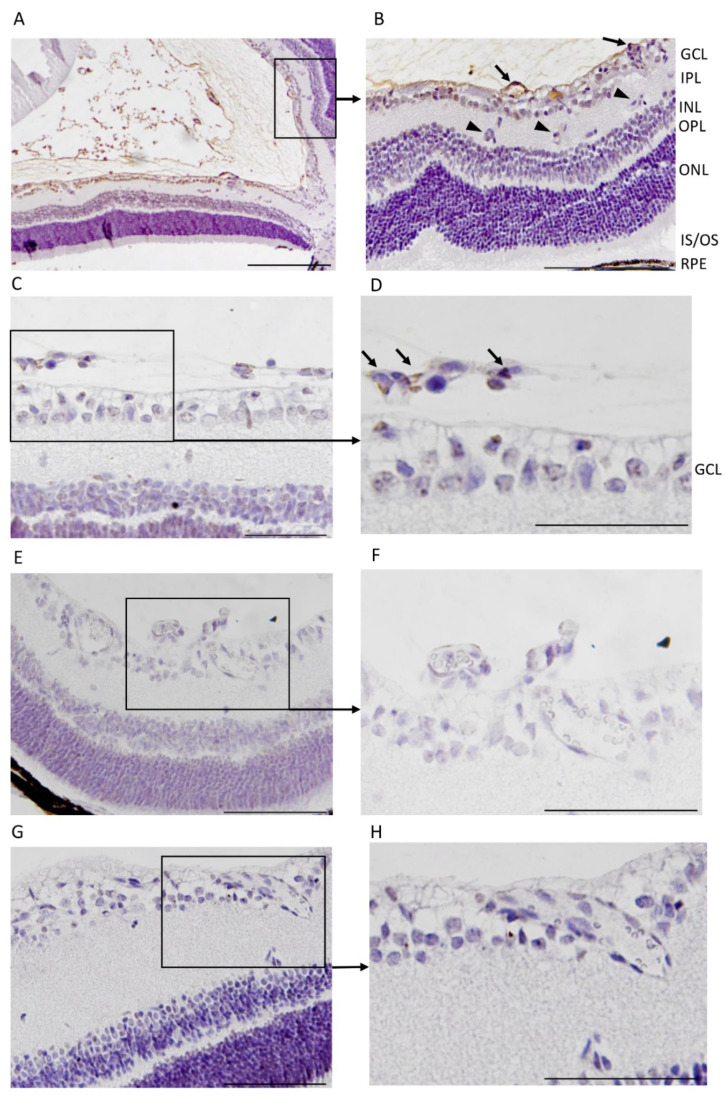
CAR-peptide (in brown) homes to pathological neovasculature in the mouse oxygen-induced retinopathy (OIR) model. Immunohistologically stained (for the detection of fluorescein in the peptide) cross sections of whole eye reveals that the systemically injected (intraperitoneal) CAR peptide (**A**–**D**) homes into the pathological preretinal blood vessels (arrows) whereas some of the normal blood vessels in the retina are not targets for CAR homing (arrowheads). Less homing was seen in animals injected with the *m*CAR-peptide (**E**,**F**), while no CAR peptide accumulation was detected in a normal (normoxia) retina after the systemic administration of the peptide (**G**,**H**). (**D**) GCL, ganglion cell layer; IPL, inner plexiform layer; INL, inner nuclear layer; OPL, outer plexiform layer; ONL, outer nuclear layer. (*N* = 12 retinas in two separate experiments). Scale bars represent 100 µm (**A**) and 200 µm (**B**–**H**).

**Figure 2:**
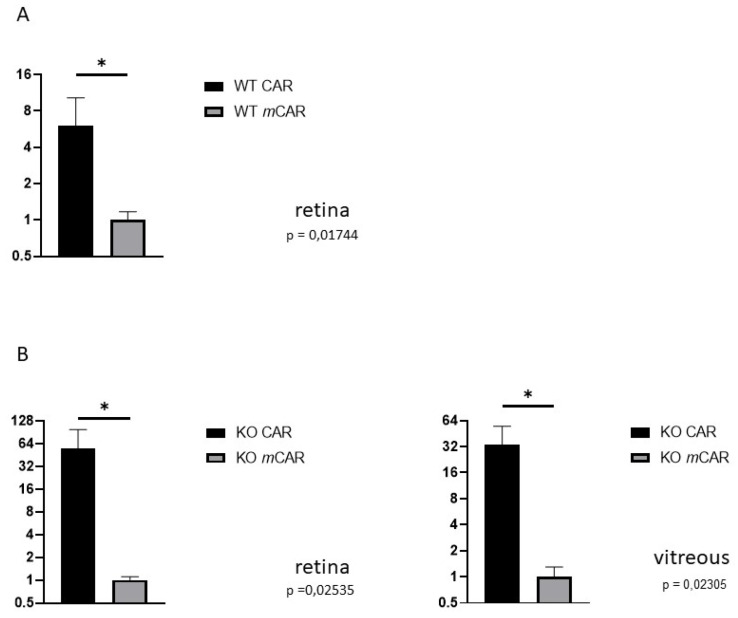
CAR peptide homes to the retina in retinopathy. The CAR and control peptide, *m*CAR, were administered intraperitoneally in normal (**A**) and R-Ras KO mice (**B**) in OIR. The homing of the peptide to the retina and vitreous was detected immunohistochemically and quantified with automated image analysis system. (**A**) CAR peptide homes to the retina 6.2-fold more than does the *m*CAR control peptide in OIR. (**B**) The angiogenic vasculature is hyperpermeable in R-Ras KO mice in OIR. The homing of the CAR peptide is increased by almost 60-fold more than the *m*CAR control peptide in OIR retina and also accumulates significantly more in the vitreous than *m*CAR in R-Ras KO mice (* *p* < 0.05).

To induce OIR, mice are placed in a 75% hyperoxic chamber at postnatal day 7 (P7) for five days, after which they are returned to normal room air [23,33]. Upon return to normoxic conditions, the avascular retina becomes hypoxic triggering revascularization of the retina [1,33]. Due to excessive hypoxic stimuli, some of the retinal blood vessels start to sprout towards the vitreous, forming pathological preretinal neovessels. We labeled CAR (amino acid sequence: CARSKNKDC) and a mutant CAR peptide (*m*CAR; where CA**R**S**K**NKDC is mutated to CA**Q**S**N**NKDC, which abolishes the homing activity almost completely) [25,26,37]) with fluorescein (FAM) and analyzed their homing to the retina after intraperitoneal (IP) administration in the OIR mice. P17 was selected as the analysis point as the angiogenesis peaks at this point in OIR [1]. Using an anti-fluorescein antibody [25,26,29,34,35]), we detected a strong signal from preretinal tufts in animals injected with the CAR peptide and the retinal signal was substantially lower for *m*CAR (Figure 1). Furthermore, the CAR peptide also penetrated outside of the blood vessels into the retina (Figure 1). We quantified the area of positive staining in the retina. The staining from the CAR peptide was 6.0-fold more than that of *m*CAR (*p* = 0.017, Figure 2).

### 3.2. CAR Peptide Homes to Bronchopulmonary Dysplasia in OIR Mice

**Figure 3:**
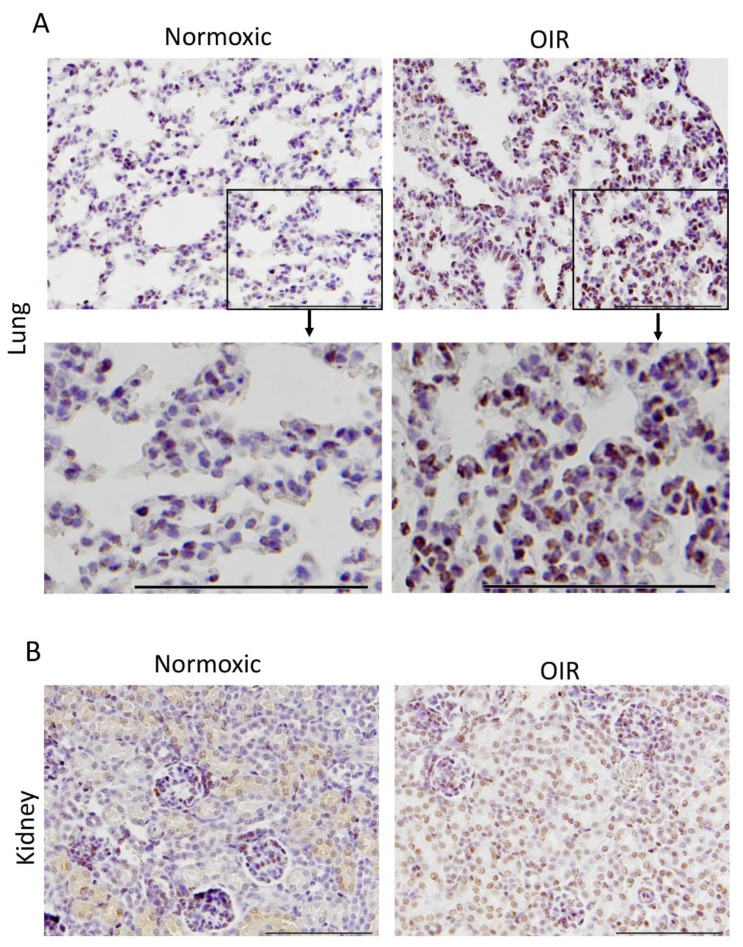
CAR-peptide homes to the lungs of OIR mice. Lung (**A**) and kidney (**B**) cross sections from normoxic and OIR mice were stained againts FITC to detect the fluorescein-labeled CAR-peptide. Strong CAR-peptide homing was seen in the bronchopulmonary dysplasia which was induced in the OIR animals, adapted from [27], published by Elsevier, 2020. The CAR peptide was seen in the kidney tissue in both normoxic and OIR animals. (*N* = 6 in OIR and 4 in normoxia). Scale bars represent 100 µm.

A large number of studies have demonstrated that the CAR peptide does not home to normal tissues [25,29,34,37,38,39,40,41]. As a small peptide it is secreted out of the body through the kidneys. In line with that data, we could not demonstrate homing to control organs in normal mice, except for strong signal related to peptide secretion from the kidney glomeruli. In addition to causing OIR in the retina, hyperoxia-/hypoxia-treatment induces an inflammatory disease, bronchopulmonary dysplasia, in the lungs [27]. Previous studies have demonstrated the homing potential of the CAR peptide to pulmonary diseases with a clear inflammatory component [29,34,38,39,40,41,42,43,44]. In line with these studies, we found that the CAR peptide homes to the bronchopulmonary dysplasia that is induced in the lungs of OIR mice (Figure 3), whereas we could not demonstrate any peptide accumulation in the lungs of mice injected with the *m*CAR peptide injected mice.

### 3.3. CAR Homes to Pathological Angiogenesis in OIR Induced in R-Ras Knockout (KO) Mice

**Figure 4:**
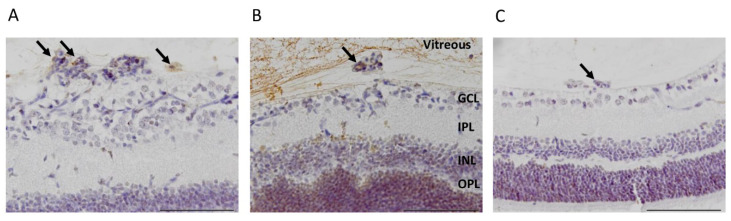
CAR-peptide homes to the preretinal tufts of R-Ras KO animals and accumulates in the vitreous. CAR-peptide (in brown) was seen in the preretinal tufts (**A**) and strong accumulatio was seen in the vitreous of R-Ras KO animals (**B**). Less CAR-peptide was seen in the vitreous of WT animals (**C**). (*N* = 2 for KO and 6 for WT). Scale bars represent 100 µm. The arrows are pointing to the CAR peptide in the preretinal tufts, i.e., pathological neovessels.

The small GTPase R-Ras has emerged as a master regulator of vascular permeability in angiogenesis [30,45,46]. We have demonstrated that the lack of R-Ras (R-Ras knockout (KO)) leads to aberrant angiogenesis in OIR [46]. The OIR induced neovascularization leads to substantially more leakage in R-Ras KO than in WT mice [46]. Thus, we used R-Ras KO mice as a “severe” retinopathy-model in OIR and performed CAR peptide homing studies in OIR with R-Ras KO mice in the same fashion as in WT mice. The CAR peptide homed to the pathological vascularization and accumulated to the vitreous with the leakage (Figure 4). Substantially more CAR peptide (55.5-fold) than control peptide was detected in the blood vessels in the retina of R-Ras KO mice in OIR (*p* = 0.025, Figure 2 and Figure 4). As the blood vessels are permeable in R-Ras KO in OIR, we also quantified the amount of peptide that had accumulated in the vitreous. The CAR peptide accumulated by a fold increase of 33.7 compared to the control peptide, *m*CAR, in the vitreous space in R-Ras KO mice in OIR (*p* = 0.023, Figure 2 and Figure 4).

### 3.4. CAR Peptide Penetrates into the Retina in OIR

**Figure 5:**
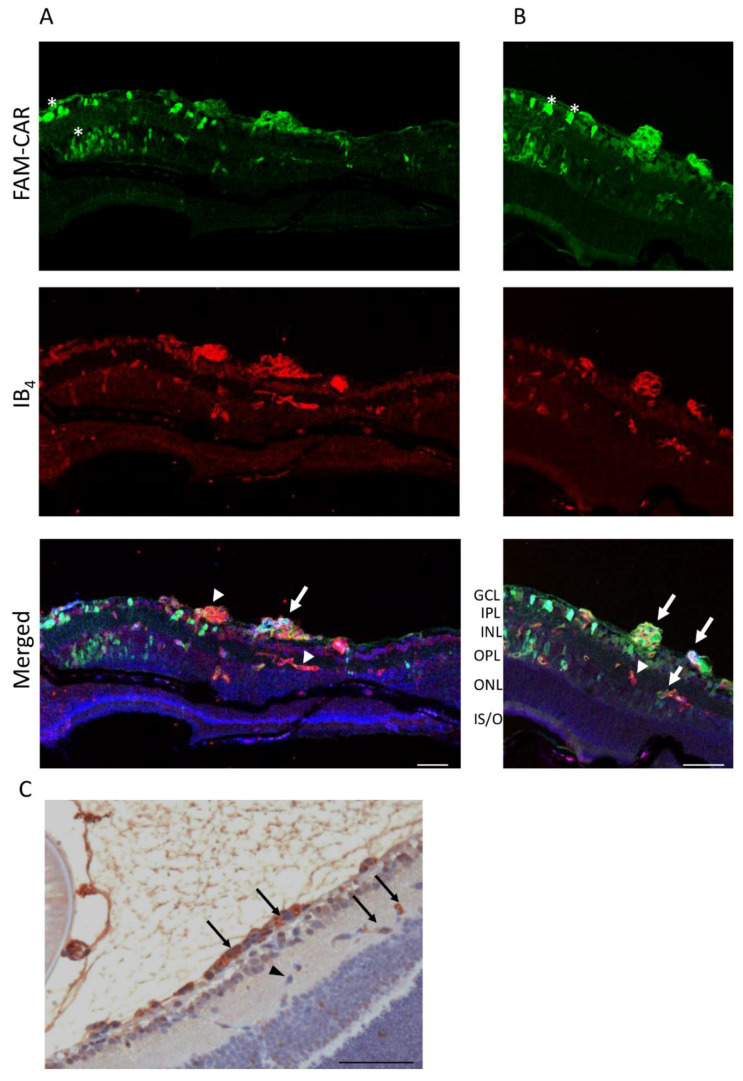
Intravitreally administered CAR-peptide binds to preretinal tufts and to the blood vessels deeper in the retina. The CAR peptide was administered intravitreously. Whole eye cross sections were stained with Isolectin B_4_ to label the blood vessels (in red) and the FAM-conjugated CAR-peptide is visible in green (**A**,**B**). The CAR-peptide was seen in the blood vessels (arrows) in different retinal layers, while some of the retinal blood vessels remained negative for CAR homing (arrowheads). CAR-homing was also seen in the neural retina, especially in GCL and INL layers (asterisks). Retina cross sections were also stained with anti-FITC antibody and the immunohistochemical signal was imaged by light microscope (**C**). The staining revealed strong CAR peptide homing/binding to preretinal neovascular tufts, but also some blood vessels in the deeper retina (arrows (**C**). (*N* = 2) GCL, ganglion cell layer; IPL, inner plexiform layer; INL, inner nuclear layer; OPL, outer plexiform layer; ONL, outer nuclear layer; IS/OS, inner and outer segments. Scale bars represent 50 µm (**A**–**C**).

Local, i.e., intravitreous (IVT), drug administration is among the most commonly used drug delivery route in treating retinal diseases. However, there are concerns that locally administered large molecules such as antibodies cannot penetrate the blood-brain-barrier and retinal layers. The CAR peptide is a cell- and tissue-penetrating peptide. Therefore, we next examined how the IVT administered CAR peptide can penetrate to retina in OIR. The CAR peptide was administered IVT and allowed to penetrate the retina for 2 h in OIR at P17. A strong signal was detected in some of the retinal blood vessels also in deeper retinal layers as well as in all layers of the retina, demonstrating cell- and tissue penetration for CAR peptide in OIR (Figure 5).

## 4. Discussion

In this study, we demonstrate the selective targeting of OIR in the retina and in bronchopulmonary dysplasia by a systemically administered vascular homing peptide, CAR. We found that the cell and tissue-penetrating CAR peptide is able to penetrate into the retina in OIR.

The CAR peptide was originally identified through the screening of phage libraries in vivo (phage display) for peptides that home to skin wounds and transected tendon injuries [25,26]. The CAR peptide was effective in homing to tissue injuries undergoing repair (skin wounds and tendon ruptures) and was shown to recognize angiogenic blood vessels at the early phase of the regenerative response [25,26]. This prompted us to test whether the CAR peptide homes to angiogenic blood vessels that form in the retina in OIR. We demonstrate homing of the CAR peptide to OIR neovasculature at the peak of angiogenesis. More recently, studies have demonstrated that the CAR peptide also homes to diseases with a clear inflammatory component such as PAH [29,34,38,39,40,41,42,43,44,47,48], vascular (aorta) aneurysms [35], muscular dystrophies [37] and myocardial infarction [49]. Due to its homing and tissue penetrating capabilities, CAR peptide has been used as a targeting device for a large number of different therapeutics in different experimental pulmonary disease models [29,34,38,39,40,41,42,43,44,47]. Recently, it was demonstrated that the hyperoxia- exposure used to induce OIR in the retina also causes simultaneous bronchopulmonary dysplasia in the lungs [27]. Accordingly, we demonstrate in this study that CAR peptide does not home to normal lungs but homes to lungs after the induction of OIR by an exposure to a hyperoxia chamber. This indicates that bronchopulmonary dysplasia could be one of the pulmonary diseases targeted by the CAR peptide.

The CAR peptide requires heparan sulfate proteoglycans (HSPGs) for its cell binding and penetrating activity [25,26]. CAR contains a homologous heparin binding site with the bone morphogenetic protein 4, and CAR binds to heparan sulfate and heparin (to their glycosaminoglycan (GAG) side chain) [25]. Although HSPGs are ubiquitous, the selective homing of CAR to angiogenic and inflammatory vasculature suggests that biological factors such as a selective expression of its receptor on angiogenesis and GAG sulfation pattern that creates a molecular signature characteristic of regenerating tissues, determine the specificity of CAR. These scenarios are actually supported by the recent finding that of the cell surface HSPGs, syndecan-4 (SDC-4) is the proteoglycan that shows substantially enhanced expression during the angiogenesis induced in the OIR model [50], whereas its expression is very low/absent in normal quiescent blood vessels [51]. Furthermore, it was also demonstrated that due to differences in the GAG chains of SDC-2 and SDC-4 (defined by a specific protein sequence in the SDC-2 ectodomain that is not present in SDC-4), the heparin-binding growth factor, VEGF, binds only to SDC-2 and not to SDC-4 [52], indicating that a specific GAG sulfation pattern is required for efficient binding.

We used OIR induced in R-Ras KO mice as a model for severe, leaky retinopathy. R-Ras is a member of the Ras superfamily of known oncogenes. However, its function is usually opposite to other members of the Ras family [53,54,55,56]; R-Ras maintains cellular quiescence and inhibits cell proliferation, whereas the other Ras family members are prominent oncogenes that can transform cells into becoming cancerous [53,57]. The generation of R-Ras deficient mice revealed the primary function of R-Ras to be blood vessel maturation and stabilization in angiogenesis [30,45,46,58,59]. Accordingly, R-Ras is considered a master regulator of vascular permeability in angiogenesis [54,60]. It is also needed for the proper lumenization of angiogenic blood vessels in hypoxia to obtain perfusion [61]. In the eye, loss of R-Ras in pericytes leads to defects in vascular supply and subsequent microphthalmia [62]. Concerning retinal diseases, R-Ras is crucial for blood vessel integrity and stabilization in the OIR-induced retinopathy [46]. R-Ras deficient mice have a hyper-permeable (leaky) phenotype in their neovessels in OIR [46]. Although the revascularization is not affected by the R-Ras deficiency, the neovessels leak twice as much in the KO mice than in the WT mice [46]. R-Ras expression in human DR samples showed that the pathological immature (VEGFR2+) blood vessels lack R-Ras expression and R-Ras expression correlates inversely with leakage from the immature blood vessels, i.e., the more leakage there is in human DR, the less R-Ras is expressed in these blood vessels [46]. Our current study demonstrates the utility of R-Ras KO mice in OIR as a model for severe, leaky retinopathy. The leakage from the pathological angiogenesis was demonstrated by the substantial accumulation of the systemically administered CAR peptide in the vitreous. Our quantitative analysis demonstrated significantly more homing of the CAR peptide to OIR, and subsequent peptide accumulation in the vitreous than of the *m*CAR peptide. We conclude that the homing and cell/tissue penetrating properties of CAR peptide (that are missing in *m*CAR) play a substantial part in the process and that the intravitreal accumulation of the CAR peptide is not caused by passive leakage.

The most significant advance in the field of targeted therapeutics is the discovery that systemically administered drugs can also be converted into target organ/disease specific forms by co-administering the drug simultaneously with certain vascular homing peptides without the need to chemically couple the peptide to the drugs [19,29,63,64,65,66,67,68]. The mechanism is the activation of a trans-tissue transport pathway by the vascular homing peptide that is capable of cell and tissue penetration [66,69,70]. The co-administered drug is essentially “swept” into this pathway and transported into the target tissue of the homing peptide (“bystander effect”). The CAR peptide is one of the rare homing peptides capable of inducing “bystander effect” on simultaneously administered drugs [29]. The CAR peptide induced the bystander effect on hypertension drugs in pulmonary arterial hypertension (PAH) and provided unique tissue selective (pulmonary) vasodilation in PAH [29]. The pulmonary vasodilation was related to the increased accumulation of anti-hypertensive drugs in the pulmonary vasculature by the CAR peptide [29]. Here we demonstrate retinal tissue penetration by the IVT administered CAR peptide. The retina itself as well as the blood retina barrier pose a significant block for drug administration [1,14,15]. This is especially relevant for large molecules such as antibodies whose therapeutic value could be hindered by poor penetration into the retina. The homing to OIR and penetration into the retina by the CAR peptide call for future studies to assess the potential therapeutic value (bystander effect) the CAR peptide might offer for drugs that are hampered by their reduced access to the retina.

## 5. Conclusions

We show that the vascular homing peptide CAR is targeted to the pathological angiogenesis in OIR and also homes to bronchopulmonary dysplasia. The selective targeting of the pathological angiogenesis in OIR and the potent tissue penetration in the retina afforded by the CAR peptide may offer entirely new opportunities to treat retinopathies effectively and with few side effects.
